# Silver Nitrate—Its Use in Dentistry

**Published:** 1898-01

**Authors:** H. Prinz

**Affiliations:** St. Louis, Mo.


### Silver Nitrate—Its Use in Dentistry.

BY H. PRINZ, D.D.S., ST. LOUIS, MO.

Pharmaceutically, there are three known preparations of the nitrate of silver.

First—Argenti nitras—Silver nitrate, colorless, rhombic plates, odorless, having a bitter, caustic metallic taste.

Second—Argenti nitras dilutus—Diluted silver nitrate, mitigated lunar caustic; white sticks formed by melting together thirty parts of silver nitrate and sixty parts of potassium nitrate and cast in molds. Besides this official preparation, there are two other varieties found in commerce containing 50 resp. 67 percent of the silver nitrate.

Third—Argenti nitras fusus—Molded silver nitrate—Lunar caustic. Hoellenstein (German). White, hard, solid sticks, fibrous fracture; formed by fusing the crystaline silver nitrate with four percent official hydrochloric acid for adding more strength to the pencil-shaped sticks.

All the foregoing preparations should be kept in dark-colored, glass-stoppered bottles to protect against direct sunlight and organic matter. For dental purposes the crystaline form or the lunar caustic is most suitable; sometimes aqueous solutions are employed, although the latter form will not keep well; the frequent opening of the bottle admits organic matter from the air and the silver will be reduced to the black oxide, rendering the solution inactive. Extemporously, the chemical might be prepared by applying a small quantity of nitric acid to the affected part of the tooth, on which quickly the end of a silver wire is rubbed. A further convenient way of application is to fuse (at 392° F.) a few crystals of the salt on the looped end of a platinum wire. Pierce recommends small pieces of blotting paper which have been previously saturated with a forty percent solution of the chemical and then dried. Again we repeat, all these preparations must be kept protected from light and air.

Stebbins (*Internat. Dent. Jour.*, Oct., '90) advocates the silver nitrate for arresting decay of the teeth. This seems to be the first mentioning of its use, but it has been used previous to that time by general practitioners as the polemic controversy between Drs. Taft and Truman (*Ibid.*) brought out. Stebbins reports about sixty-four cases of caries, in which he used the silver salt. Inside of one year thirty-seven did not show any new decay, in fourteen cases he found minute starting points, and in thirteen cases he could not show any success. He applied the caustic to the superfacial buccal cavities and other invisible places in bicuspids and molars, but specially in deciduous teeth by using very small crystals, which were brought into the moistened cavity. The adjacent parts had to be protected by a napkin. The only objection against the silver-nitrate is the discoloration of the applied parts, the reduced silver forms a black deposit in the dental canals, and for this reason it is contra-indicated in the treatment of the anterior teeth.

In sensitive dentine, specially in cases where the gums have retracted, as in senile age, or where the clamps of artificial dentures have caused abrasion, silver nitrate is of most efficient value. By painting the affected part with lunar caustic, in two or three sittings the hypersensitiveness will usually subside. In superfacial cavities, which often can not bear the touch of an excavator, it is also of great merit. Deposits of food and mucus should be removed, a drop of water applied, and then a small crystal of the salt is gently rubbed over the moistened surface. Any remaining liquid should be absorbed by cotton, and, if possible, the cavity filled temporarily with cement. In two or three weeks we will find with astonishment the previous sensitiveness entirely vanished (Miller).

Silver nitrate is the ideal escharotic. When brought in contact with living tissue, a dense film of coagulated albumen results, but owing to this film, the depth of the action is very limited. The albuminous coating is at first white, but soon becomes blackish, owing to the reduction of the silver. (U. S. Disp.) Aphthous ulcers, hypertrophic gums, badly healing wounds (after extraction), papilloma, etc., will be greatly benefited by the judicious application of the drug, either in substance or in solution.

